# Supplementary material for: Particulate matter 10 induces oxidative stress and apoptosis in rhesus macaques skin fibroblast
Source: PeerJ. 2023 Dec 18;11:e16589. doi: 10.7717/peerj.16589 (PMC10734408; doi:10.7717/peerj.16589)
Supplement: Supplemental Information 1 [file peerj-11-16589-s001.docx]

**Supplementary Table**

**Supplementary Table 1. Composition of Particulate matter 10**

| **Fine Dust (PM10-Like)** | | | | |
| --- | --- | --- | --- | --- |
| **Element** | **Mass Fraction** | | | |
|  | **Certified value  (mg/kg)** | | **Uncertainty  (mg/kg)** | |
| Arsenic | 7.1 | | 0.7 | |
| Cadmium | 0.9 | | 0.22 | |
| Lead | 113 | | 17 | |
| Nickel | 58 | | 7 | |
| **Additional Material Information** | | | | |
| **Element** | **Mass Fraction** | **Element** | | **Mass Fraction** |
|  | **Value (mg/kg)** |  |  | **Value (mg/kg)** |
| Aluminium | 34100 | Molybdenum | | 33.2 |
| Antimony | 64.7 | Neodymium | | 22.2 |
| Barium | 562.2 | Potassium | | 10998 |
| Bromine | 10.2 | Rubidium | | 52.3 |
| Caesium | 3.1 | Samarium | | 4.1 |
| Calcium | 63043 | Scandium | | 7.4 |
| Cerium | 56.8 | Silicon | | 229000 |
| Chlorine | 10033 | Sodium | | 14211 |
| Chromium | 201 | Strontium | | 251 |
| Cobalt | 14.3 | Tantalum | | 1 |
| Copper | 462 | Terbium | | 0.6 |
| Dysprosium | 3.3 | Thorium | | 7 |
| Elemental carbon | 45433 | Titanium | | 4372 |
| Europium | 0.8 | Total carbon | | 111333 |
| Gallium | 8.7 | Total organic carbon | | 76633 |
| Gold | 0.02 | Tungsten | | 4.1 |
| Hafnium | 8.4 | Uranium | | 2.6 |
| Iron | 38144 | Vanadium | | 72.4 |
| Lanthanum | 25 | Ytterbium | | 1.7 |
| Magnesium | 13200 | Zinc | | 1240 |
| Manganese | 611 | Zirconium | | 341 |

**Supplementary Table 2. Primers used for qPCR**

| **Gene Name** | **Genebank Number** | **Product size(bp)** | **Primer sequence** |
| --- | --- | --- | --- |
| *GAPDH* | NM_001195426.1 | 111bp | F : aaggtagtgaagcaggcgtc  R : gtcgaaggtggaagagtggg |
| *BAX* | NM_001261016 | 114bp | F : aaggtgcccgaactgatcag  R : aaagtaggagaggaggccgt |
| *CASP3* | XM_015139582.2 | 109bp | F : tgcatactccacagcacctg  R : ttcaagcttgtcggcgtact |
| *CYCS* | XM_028845949.1 | 100bp | F : cgttgaaaagggaggcaagc  R : attggcggctgtgtaagagt |
| *TP53* | NM_001047151.2 | 102bp | F: cgcttcgagatgttccgaga  R: cttcaggtggctggagtgag |
| *GPX1* | NM_001159298.2 | 108bp | F : cttcccgtgcaaccagtttg  R : agcatgaagttgggctcgaa |
| *GPX3* | NM_001159300 | 142bp | F: aggtggaggctttgtcccta  R: ccagaagaggcggtcagatg |
| *CAT* | XM_001115625.4 | 134bp | F : gaccactggagctggtaacc  R : gctcgggaattctctctcgg |
| *NDUFA1* | NM_001257292 | 121bp | F: cgtgtgcttgcttattccagg  R: gcgcctatctctttccatcagat |
| *NDUFA2* | NM_001261499 | 149bp | F: tcgaggaataggggcaaagc  R: cgcggattaggatgggtagg |
| *NDUFC2* | NM_001266174 | 100bp | F: gaacccagagcccttacgg  R: gtatcccaagaagccgacgt |
| *NDUFS4* | XM_001096347 | 104bp | F: gttgtggcgaagggtagtgg |
|  |  |  | R: cttgagtctggtcctgtgcc |
| *ATP5H* | NM_001193605 | 149bp | F: acctgagactccaccagcta  R: tttcttcggcatccacctgg |
|  |  |  |  |
